# Multiscale consensus habitat modeling for landscape level conservation prioritization

**DOI:** 10.1038/s41598-020-74716-3

**Published:** 2020-10-20

**Authors:** Erin E. Poor, Brian K. Scheick, Jennifer M. Mullinax

**Affiliations:** 1 Department of Environmental Science and Technology, University of Maryland, 1433 Animal Science Building, 8127 Regents Dr., College Park, MD 20742 USA; 2 Fish and Wildlife Research Institute, Florida Fish and Wildlife Conservation Commission, 1105 SW Williston Rd., Gainesville, FL 32601 USA

**Keywords:** Conservation biology, Climate-change ecology, Ecology, Ecological modelling

## Abstract

Globally, wide-ranging carnivore populations are imperiled due to human-caused habitat fragmentation. Where populations are fragmented, habitat quantification is often the first step in conservation. Presence-only species distribution models can provide robust results when proper scales and data are considered. We aimed to identify habitat for a fragmented carnivore population at two scales and aid conservation prioritization by identifying potential future habitat fragmentation. We used location data and environmental variables to develop a consensus model using Maxent and Mahalanobis distance to identify black bear (*Ursus americanus floridanus*) habitat across Florida, USA. We compared areas of habitat to areas of predicted sea level rise, development, and protected areas. Local-scale models performed better than state-scale models. We identified 23,798 km^2^ of habitat at the local-scale and 45,703 km^2^ at the state-scale. Approximately 10% of state- and 14% of local-scale habitat may be inundated by 2100, 16% of state- and 7% of local-scale habitat may be developed, and 54% of state- and 15% of local-scale habitat is unprotected. Results suggest habitat is at risk of fragmentation. Lack of focused conservation and connectivity among bear subpopulations could further fragmentation, and ultimately threaten population stability as seen in other fragmented carnivore populations globally.

## Introduction

Globally, wildlife is now being lost faster than at any other point in history, largely due to habitat degradation and fragmentation^1–4^. Because wide-ranging large mammals, such as carnivores, occur at low population densities and require large expanses of habitat, they may be more affected by habitat loss than other taxa^5^. When human development and infrastructure fragments the landscape, carnivore populations may also be fragmented, resulting in isolated subpopulations, which may accelerate local or global extinction^6–11^.

Past declines in large carnivore populations, such as cougars (*Puma concolor*), grey wolves (*Canis lupus*), and black bears (*Ursus americanus*), have resulted in large landscapes lacking carnivores across North America. Increased education and carnivore-friendly wildland management have aided in the population recovery of some of these traditionally persecuted species, particularly the black bear. However, while many local black bear populations may be increasing^12–14^, these population increases are not consistent across the species’ range. Populations in southeastern and southwestern U.S. and Mexico remain fragmented^15^, with uncertain future population trajectories. Recovery of all isolated subpopulations and subspecies, such as the genetically distinct Florida black bear (*Ursus americanus floridanus*), can help increase genetic diversity of the species as a whole, thus safeguarding the population from future environmental heterogeneity due to direct human impacts or climate change^16^.

**Figure 1:**
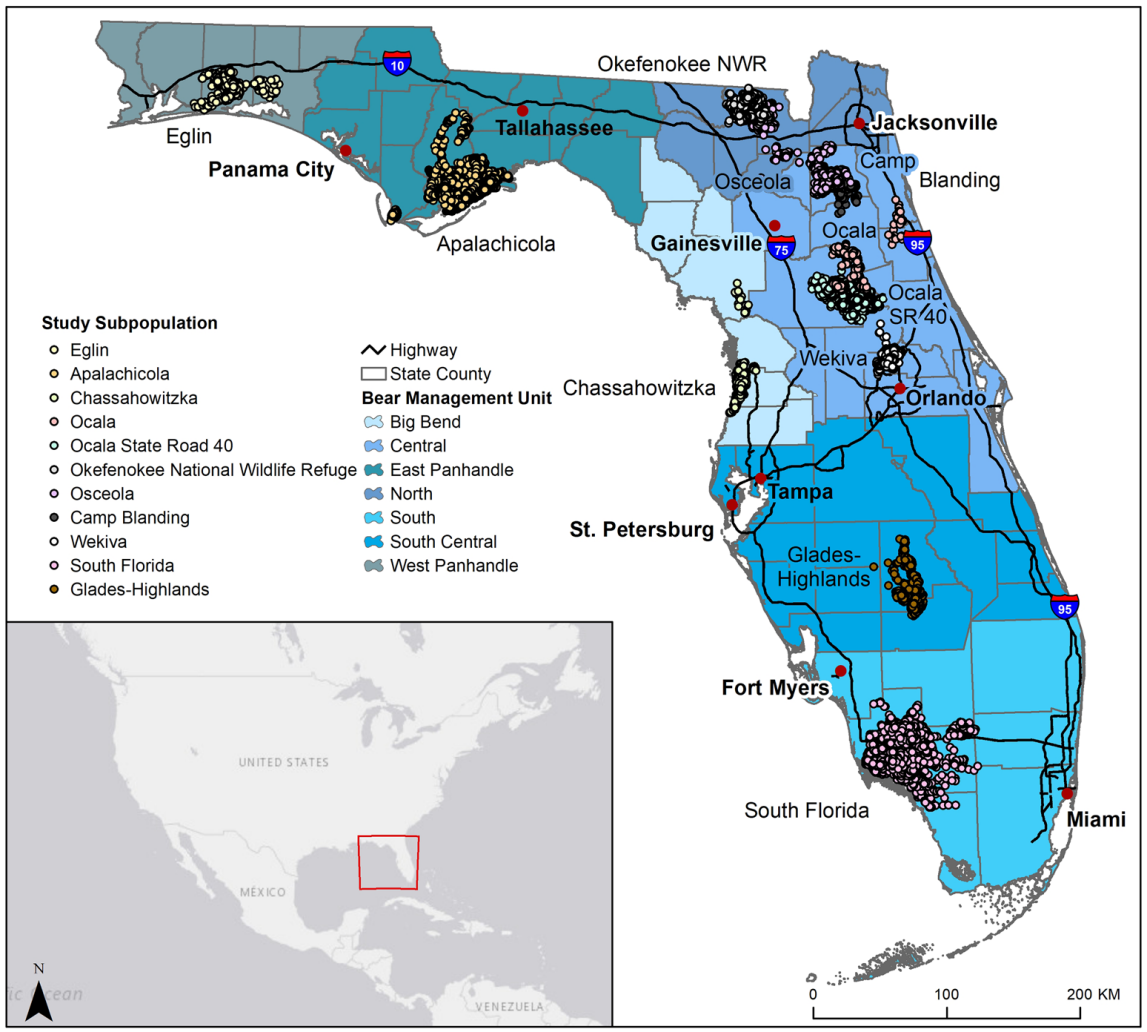
Florida black bear (*Ursus americanus floridanus*) locations. Location of study bear locations, major roads, cities and bear management units (BMUs) throughout Florida. Created using ArcMap 10.4 (Esri 2015).

The Florida black bear originally ranged throughout Florida and the southern portions of neighboring states^17^. The estimated pre-European settlement bear population in Florida was ~ 11,500 individuals^18^, but the black bear population began to decline after European colonization, largely from direct persecution and extensive land clearing. Since 1974, when Florida listed the black bear as Threatened, the bear population has been slowly increasing. Yet still today, the statewide range covers only half of what it once did^19^, distributed in several distinct and recently reconnected areas (Fig. 1;^19,20^). These subpopulations vary in size from ~ 18 to 1198 individuals^21,22^ with a recent statewide estimate of ~ 4000 bears^19^. Differences in subpopulation size and density are likely due in part to naturally occurring differences in food availability and distribution across a biologically diverse state. To better manage such inherent diversity, the Florida Fish and Wildlife Conservation Commission created bear management units (BMUs) in 2012 (Fig. 1;^23^) based on geographic commonalities and human population distribution, and the likely impact of those characteristics on bear management. Black bear dispersal is naturally restricted by the peninsular geography of Florida, and future sea level rise may threaten dispersal and habitat distribution for coastal subpopulations. Dispersal and habitat will likely be further threatened in the future by significant human development, as Florida has the third largest and third fastest growing human population in the U.S.^24^.

Identifying and protecting existing habitat is often one of the first steps of wildlife conservation. For carnivores, identifying habitat at a sufficiently large scale (such as at the population or subpopulation level) can be problematic due to inconsistencies or lack of data, differing regional wildlife management or policies, or lack of specific knowledge of habitat requirements^25,26^. Furthermore, species absence information is usually lacking, given the large home ranges and long dispersal capabilities in sometimes seemingly sub-par habitat^27^. As such, presence-only species distribution models (SDM) that indicate habitat suitability across individuals throughout populations^28^ have been frequently used. Stand-alone presence-only models have come under some scrutiny due to their sensitivity to inputs, scale, and accuracy measures^28–30^. Nevertheless, these models can provide robust information about habitat distribution, especially when multiple models are used to identify a habitat ‘consensus’ and thus limit the uncertainty due to one specific model type^31,32^. Furthermore, using presence-only models at multiple scales, such as at a landscape scale and at a localized scale, allows identification of population and subpopulation level species distributions and subpopulation-specific requirements based on local environmental variability^33,34^.

Landscape scale habitat conservation for the fragmented bear population in Florida requires knowledge of habitat distribution within a broader context, beyond habitat selection at the individual scale as provided by previous resource selection studies. While local, home range habitat selection has been studied for most subpopulations in Florida^35–40^, statewide habitat identification using robust multivariate quantitative methods has not been completed. Thus, as a major step in the statewide black bear habitat conservation effort, we aim to identify and quantify Florida black bear habitat (1) at the landscape scale across all of Florida, and (2) at the local, subpopulation scale, using the BMU extent, by applying consensus presence-only SDMs. For conservation prioritization, we then identify areas of suitable habitat that will be threatened by projected sea level rise or by projected future development and areas that are currently protected.

## Methods

### Study area

Florida is a biodiverse state and spans four ecoregions^41^. The East Gulf Coastal Plain on Florida’s panhandle is characterized by longleaf pine-dominated uplands, pine flatwoods and savannas, and bottomland hardwood forests, with sandy, clay, and silty soils. The South Atlantic Coastal Plain, in Florida’s northeast, contains longleaf pine (*Pinus palustris*) forested flood plains with rainfall of 52–64 in per year. The Peninsular Florida ecoregion can be characterized by a temperature ranging from 23° to 95° F, approximately 65 in of rainfall per year, and heavy urbanization in the Tampa and Orlando areas, with some hardwood forests. Finally, South Florida has a true tropical climate, with temperatures ranging from 47° to 90° F, an average of 60 in of rainfall per year, large areas of agriculture and urbanization, and most of the remaining scrub oak, sand pine, and Everglades ecosystems.

The diverse, unique ecosystems of Florida have been heavily impacted by the human population. Florida has the eighth highest human density of any state in the United States, (~ 136 people/km^2^), the third largest growth rate, and the third largest population in the United States, with projected growth to reach 23 million by 2030^42^. Florida’s Growth Management Act (GMA), implemented in 1985, is recognized as one of the nation’s ‘best practices’ in an attempt to curtail sprawl^43^. However, the GMA has inadvertently resulted in an increase in housing in suburban and rural areas^44^. Consequently, development of natural, agricultural, and rural areas continues. The Florida 2070 Project recently created development scenarios under a business-as-usual scenario and an alternative scenario with more compact development. Under the business-as-usual scenario, 1/3 of Florida’s land will eventually be developed for human use in a low-density pattern^24^.

### Species data

We used historic, non-systematic locations of black bears collected via VHF and GPS collars from a variety of researchers from 1983–2018 (Fig. 1)^17,35,36,38,39,45–53^. We screened the data and removed bears < 4 years old because we were interested in modeling habitat for resident adults, not juvenile dispersers^54^. We also removed bears with fewer than 30 locations within a 12-month window or with locations collected across < 3 months in a 12-month period. From the remaining bears, we removed all GPS locations with low precision as indicated by fix status, or position dilution of precision > 7^55,56^, as well as all capture and mortality locations. To reduce spatial autocorrelation and bias from differing fix frequencies between individuals and collar types and to retain information on multiple locations at different times per day, we subsampled GPS collar data to locations every 5 h^57–60^. We used R package amt^61^ to sub-sample the GPS data. Because we were interested in identifying general black bear habitat, we combined male and female bears, as well as all seasons.

### Environmental data

We created 17 habitat variables based on a literature review of Florida black bear subpopulations (Supplementary Material Table 1)^17,37,39,40,48,62,63^. Variables represented characteristics of vegetation, water, anthropogenic features, and topography. Where we calculated local density variables, we used 0.5 km as the moving window radius, based on the average daily movement of a female black bear^64^, because we wanted to capture habitat features that might be directly available to any bear.

We included several measures of natural vegetation and forage-specific vegetation^17,62^. We defined “natural” vegetation as any vegetation not identified as agricultural, urban, or suburban as defined by the Florida Cooperative Land Cover Dataset^65^ and “forage” vegetation as that identified by state bear biologists as important food sources for bears (Supplemental Material Table 1). We calculated Euclidean distance to natural vegetation and forage vegetation, forage vegetation neighborhood (1.5 km radius moving window) and local (0.5 km) density, and area-weighted natural vegetation contiguity and shape area index in Fragstats v. 4^66^. Shape index was the normalized ratio of edge to area compared to a square patch. A value of 1 indicated a square patch, and > 1 indicated a more complex patch shape. Patch contiguity was calculated by weighting orthogonally connected patch cells and summing across a local window (0.5 km^2^).

We included topographic ruggedness index (TRI)^67^ and elevation^68^ as measures of topography from the 2016 National Elevation Dataset at 30 m resolution^69^. We calculated TRI by taking the square root of the average squared differences in elevation from a center pixel and its eight neighboring elevation pixels^67^. Higher values of TRI indicated areas that were more rugged.

Florida black bear subpopulations vary in their use of agriculture^39,46^. Use and avoidance of these areas likely depends on a variety of factors, including sex, agriculture type and availability on the landscape, and available land cover alternatives^70^. We tested two different measures of agricultural areas: density of agricultural patches and Euclidean distance to agriculture. We used the USDA National Agricultural Statistics Service 30 m raster to identify agricultural areas^71^.

Areas of urban development and roads can negatively impact black bear habitat selection, movement, and survival^39,72^ and we included population density^73^ and distance to nearest major city center. Because bears may use areas of varying road densities and traffic volumes differently^74^, we included primary, secondary, and tertiary roads as separate road density variables, derived from U.S. Census Bureau TIGER/Line 2016 data^75^. Primary and secondary roads were defined as highways, interstates, and major roads (S1100 and S1200 MTFCC) and tertiary roads were defined as local neighborhood roads, rural roads, city streets, and smaller roads (S1400, S1500, S1640, S1710, S1730, S1740, S1820, S1830 MTFCC).

Riparian zones, swamps, and creeks can positively influence bear habitat selection^39,48^. Therefore, we included the density of rivers and flowlines (e.g., rivers, creeks, canals) as defined by the National Hydrology dataset^76^ as well as density of freshwater forested and shrub wetland patches. We used the National Wetlands Inventory^77^ to identify all other wetland areas.

Variables were created at or rescaled to 120 m × 120 m resolution to capture conditions within bear home ranges and daily movements^64^, projected to Florida 1983 GDL Albers, and then screened for correlation. We prepared data in R statistical software version 3.6^78^ and ArcGIS 10.4^79^.

### Habitat modeling

While location data from collars may also be used to create resource selection or step selection functions, our goal was to identify black bear habitat across the entire state. From the available bear presence data, few of the subpopulations had high-resolution GPS collar data and the VHF collar data across the state was inconsistent and infrequently collected, thus, we determined SDMs were the best method to identify population-wide habitat suitability. From the many options for presence-only habitat models^80^, we chose maximum entropy (Maxent) and Mahalanobis distance habitat suitability models^68,81,82^.

We chose Maxent due to its popularity in the literature^30^, its ease of use, and its ability to model complex relationships among covariates lacking in other methods^83,84^. Additionally, Maxent performs consistently well across ecosystems, species, and scales^32,80,85–90^. Maxent models attempt to approximate the probability of a species presence, conditioned on environmental variables, using presence-only data supplemented with model-generated background locations^83^. Simply put, conditional probability is calculated across the study area using the conditional density of transformed covariates and their interactions at the presence locations and the unconditional background locations (See Elith et al.^83^ for a complete explanation).

Mahalanobis distance modeling provides an alternative to Maxent which is more straightforward^68,91^. Mahalanobis distance is the distance measured in standard deviations in multivariate space from the value of one sample to the average of the distribution^92^. When applied to habitat suitability modeling, Mahalanobis distance is the difference between environmental covariate values across the study area and the ‘ideal’ covariate values found at the species’ locations^58^. Mahalanobis distance habitat modeling is a true presence-only model, requiring no background samples, unlike Maxent. We chose to include Mahalanobis distance here due to its prior use on bears, relative simplicity compared to Maxent, and ability to perform well across species and ecosystems^26,32,68,93,94^.

We created Maxent and Mahalanobis distance models at two scales: (1) the bear management unit (BMU), as a local-scale, to provide detailed habitat information for specific subpopulations throughout the state, and (2) at the broader state-scale, to provide a general statewide habitat identification and assessment. We restricted local models to BMU boundaries buffered by 20 km. The buffer reduced any introduced bias at BMU edges resulting from mosaicking models together.

We ran Maxent version 3.4.1 with default settings, including hinge features, jackknifing, response curves, and 10,000 random background locations^81^. We created Mahalanobis distance models using the *mahasuhab* function of the adehabitatHS R version 3.6 package, with the output type as ‘probability’^78,82^. To reduce spatial sampling bias with the creation of background locations in Maxent at the state-scale, we created a 95% kernel density estimate (KDE) utilization distribution for all filtered bear locations using the adehabitatHR package in R with the h_ref_ smoothing factor^82^. Then, we used the resulting polygon as the boundary within which Maxent selected background locations^83,95^. For Maxent and Mahalanobis model training and testing, we used tenfold cross-validation. For each model iteration, we wrote the Maxent background locations to file, for later use in model accuracy assessment. We used a modified reverse step-wise approach for model selection for both Maxent and Mahalanobis models. We first included all variables, then selected those variables that had a percent contribution > 1 and were uncorrelated, in Maxent. For Mahalanobis models, we iteratively removed variables and identified variable contributions using principal components analysis in the stats R package^78^. In Maxent models, if two correlated variables were included in the same model, we retained the variable with the higher percent contribution.

For accuracy assessment, we calculated five accuracy measures. First, we calculated two threshold-independent accuracy measures—AUC and the Boyce index^96–99^—for each of the 10 Maxent and Mahalanobis continuous model outputs for each modeling extent (seven BMUs and one statewide extent). We used the background locations created from the Maxent models to calculate AUC for the Mahalanobis outputs using the ROCR package in R^100^. We calculated the Boyce index in the ecospat R package with the default moving window for both Maxent and Mahalanobis outputs^101^. Next, we calculated three threshold-dependent accuracy measures. To do so, we used the maximum sensitivity and specificity value from Maxent iterations to select thresholds on which to base binary habitat maps^102^ of each model iteration for each extent. Using these binary maps and the background locations identified by each Maxent iteration, we calculated the true skill statistic (TSS). The TSS is a threshold-dependent accuracy measure which takes sensitivity and specificity into account, and is independent of species prevalence^29^. The TSS, like the Boyce index, ranges from -1 to 1, where 1 indicates perfect model accuracy, and 0 indicates a model no better than random^29^. Finally, to identify errors of omission and commission, we calculated sensitivity and specificity for each thresholded model output. A full list of accuracy measure results for each model iteration may be found in Supplementary Material Tables 2–9.

We then averaged the continuous outputs created from the models (for each extent 10 Maxent and 10 Mahalanobis models) to create one continuous output for each extent (seven BMUs and one statewide output). Next, we mosaicked the continuous local-scale models in ArcGIS 10.4^79^ to create one local-scale map for the entire state. We applied the averaged maximum sensitivity plus specificity value to each averaged continuous model and then mosaicked the thresholded model outputs to create a statewide binary map depicting local-scale habitat. Finally, we summed the local-scale and state-scale binary maps to create an output depicting 3 habitat categories; local-only habitat, state-only habitat, and habitat identified at both scales. For display purposes, from the continuous, averaged outputs, we created a cumulative frequency distribution from the bear locations at 10% intervals (each interval contained a cumulative percentage of the bear locations)^103,104^.

### Threats and protection

To provide conservation prioritization guidance, we identified habitat areas within future sea level rise inundation areas, areas of projected future development, and unprotected areas. We used the high certainty areas of the mean high water 30 cm and 305 cm sea level rise estimated to occur by 2100^105^ as best- and worst-case scenarios for inundation and calculated areas of overlap with the categorical habitat maps. We next overlaid the categorical maps with the Florida 2070 Project’s 2070 Development Scenario geospatial data^24^, to determine amount and distribution of habitat which may be threatened by future development. Finally, we compared the categorical output to all protected lands in Florida to determine how much habitat is not currently under local, state, or federal protection^65^. These analyses were conducted in ArcGIS 10.4^79^.

## Results

### Species and environmental data

After data screening, we included 86,604 bear locations (of 277,766) from 236 (of 262) adult bears (Fig. 1). Wetland density and TRI (r = − 0.73), river density and creek density (r = 0.73), neighborhood food vegetation density and local food vegetation density (r = 0.99) and contiguity and shape index (r = 0.99) were highly correlated and not included in the same Maxent models.

### Modeling outputs

**Figure 2:**
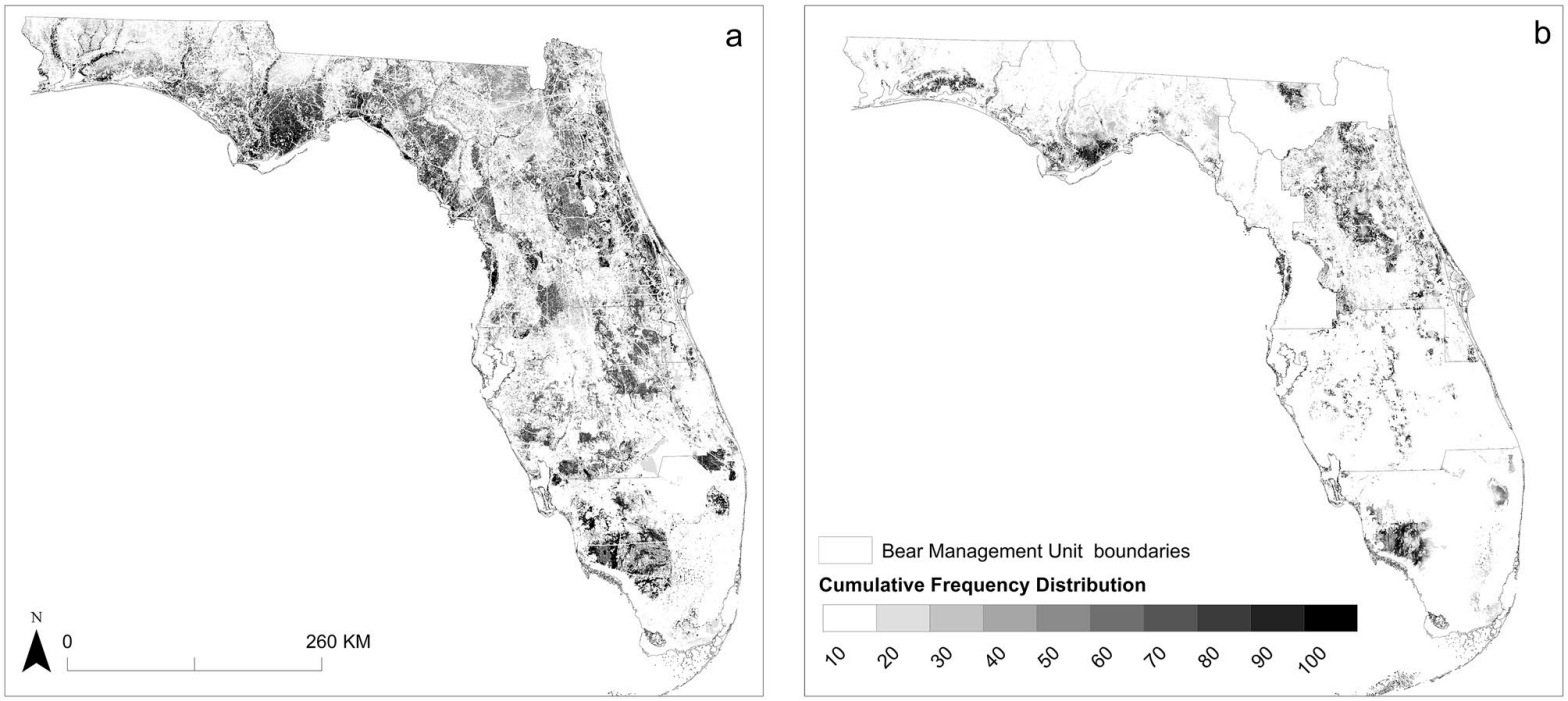
Modeled Florida black bear (*Ursus americanus floridanus*) habitat throughout Florida. Consensus model of black bear habitat suitability as modeled statewide (**a**) and at the bear management unit, or local-scale (**b**) using Maxent and Mahalanobis distance models. Cumulative frequency distribution values in 10% intervals, (each interval contained a cumulative percentage of the bear locations). For example, the 80% binned cells are 10% more likely to contain a bear location than the 70% bin and 70% more likely to contain a bear location than the 10% bin. Created using ArcMap 10.4 (Esri 2015).

**Figure 3:**
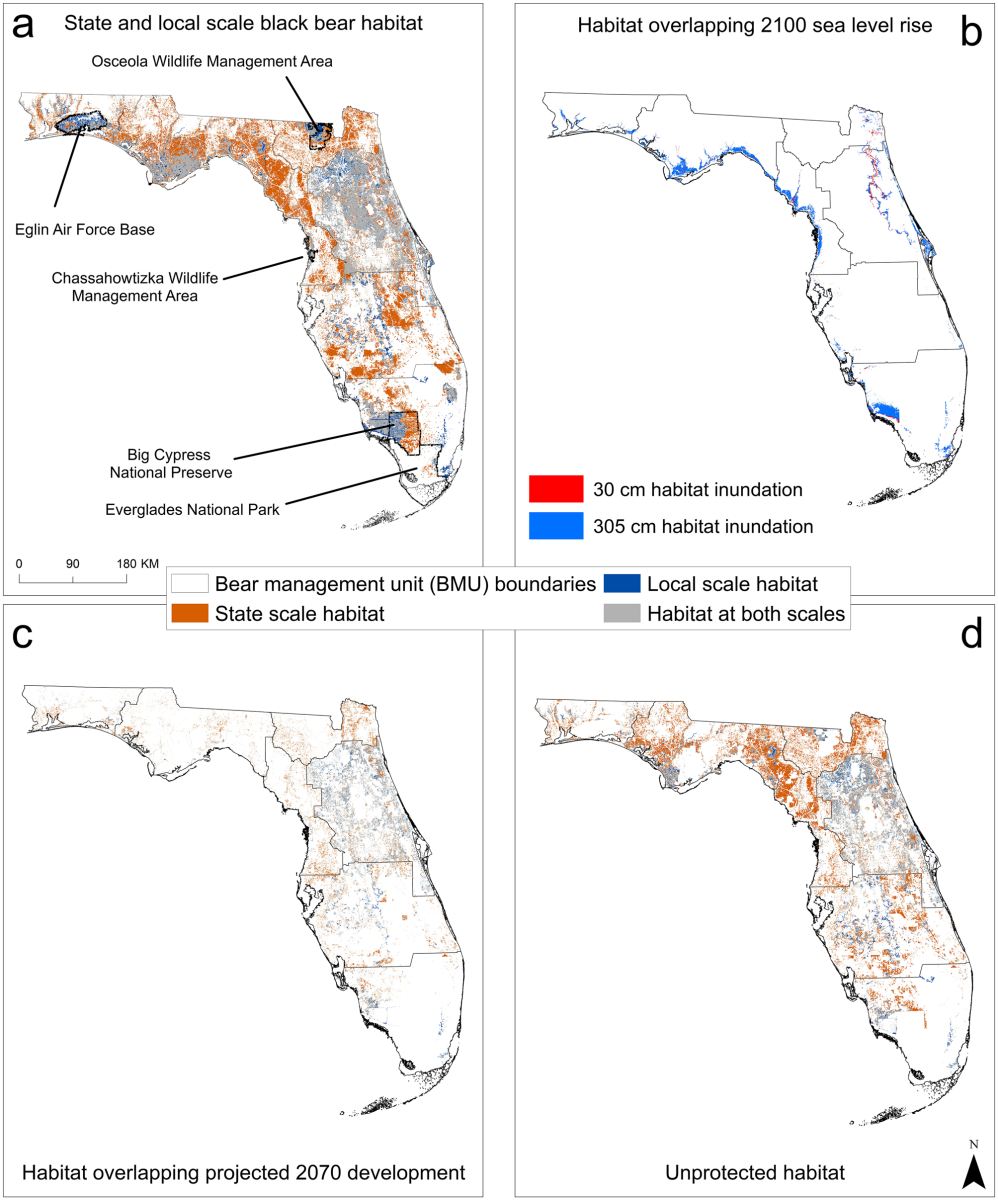
Florida black bear (*Ursus americanus floridanus*) habitat and impacts of inundation, development, and projection. Florida black bear habitat identified at the state- and local-scale using an average maximum testing sensitivity plus specificity threshold from Maxent habitat models (**a**), areas of all combined habitat potentially inundated under 30 cm and 305 cm sea level scenarios (**b**), habitat that intersects with potential development, from a 2070, business-as-usual scenario (**c**), and habitat that is not under county, state, or federal protection (**d**). Created using ArcMap 10.4 (Esri 2015).

On average, the local-scale models performed slightly better than the state-scale model, but both had relatively high accuracies (Table 1; Supplementary Material Tables 2–9). Final variable inputs and relationship direction varied by model (Table 2). Most habitat was identified along the northern gulf coast, the higher elevation areas of central Florida, and the southern Gulf Coast (Figs. 2 and 3a).

**Table 1:** Accuracy assessment measures, area under the curve (AUC), Boyce Index, true skill statistics (TSS), sensitivity, and specificity for each black bear species distribution averaged across model iterations, using two habitat suitability models, Maxent and Mahalanobis distance, for a state-scale model and for seven bear management units.

	Maxent					Mahalanobis				
	AUC	Boyce Index	TSS	Sensitivity	Specificity	AUC	Boyce Index	TSS	Sensitivity	Specificity
Big Bend	0.92	0.97	0.86	0.93	0.93	0.95	0.95	0.76	0.83	0.94
Central	0.80	0.99	0.46	0.80	0.65	0.82	0.98	0.46	0.66	0.80
Eastern Panhandle	0.84	0.98	0.56	0.81	0.76	0.93	0.99	0.67	0.76	0.91
North	0.84	0.98	0.83	0.96	0.87	0.93	0.99	0.78	0.83	0.95
South Central	0.87	0.98	0.83	0.95	0.88	0.93	0.99	0.70	0.79	0.91
South	0.82	0.99	0.78	0.91	0.86	0.9	0.98	0.62	0.74	0.88
Western Panhandle	0.91	0.95	0.76	0.93	0.83	0.86	0.9	0.57	0.82	0.75
State	0.76	1.00	0.36	0.79	0.57	0.71	0.99	0.31	0.76	0.55

**Table 2:** Variable ranks and directions (in parentheses) for black bear habitat suitability models created at a state-scale and bear management unit scale with Maxent and Mahalanobis distance modeling methods.

Variable	Big Bend BMU		Central BMU		Eastern Panhandle BMU		North BMU		South BMU		South Central BMU		Western Panhandle BMU		State-scale	
	Maxent	MD	Maxent	MD	Maxent	MD	Maxent	MD	Maxent	MD	Maxent	MD)	Maxent	MD	Maxent	MD
Agriculture density	–	–	4	–	4	–	–	–	–	8( +)	5	–	–	–	5	7( +)
Distance to agriculture	–	3( +)	–	9(−)	–	5( +)	5	2(−)	–	–	–	2(−)	–	–	–	–
Elevation	1	2(−)	–	1(−)	–	8( +)	2	9(−)	5	2(−)	2	3( +)	2	2(−)	–	5( +)
TRI	–	–	–	–	–	–	–	–	–	–	–	–	–	–	–	–
Distance to cities	–	8(−)	–	8( +)	–	3( +)	–	1( +)	4	6(−)	3	7(−)	–	7(−)	–	–
Population density	3	–	–	–	–	–	1	–	–	–	–	–	1	–	–	–
Distance to flowline	5	–	–	–	–	9(−)	–	8( +)	–	9( +)	–	–	4	5(−)	–	–
Distance to rivers	–	5( +)	–	6(−)	–	–	–	–	6	–	–	8( +)	–	–	–	6( +)
Primary road density	–	6(−)	3	–	3	–	–	–	–	–	–	–	–	–	3	8( +)
Teritiary road density	7	–	–	3(−)	–	2(−)	–	6(−)	–	5(−)	–	5( +)	5	4( +)	–	–
Distance to natural vegetation	–	7( +)	1	5(−)	–	4(−)	–	4(−)	3	1( +)	–	6( +)	–	–	1	1(−)
Natural vegetation contiguity	–	–	–	–	2	1( +)	4	3( +)	–	3(−)	4	–	–	–	–	–
Natural vegetation shape index	6	9( +)	–	7(−)	–	–	–	–	–	–	–	9(−)	–	6(−)	2	3( +)
Distance to forage vegetation	–	–	2	–	–	–	–	–	–	–	–	–	–	–	–	2(−)
Local density of forage vegetation	2	4( +)	–	4( +)	–	7( +)	–	7(−)	–	4(−)	1	4(−)	–	3(−)	–	–
Neighborhood density of forage vegetation	–	–	–	–	–	–	–	–	1	–	–	–	3	–	–	–
Wetland density	4	1( +)	5	2( +)	1	6( +)	3	5( +)	2	7( +)	–	1(−)	–	1(−)	4	4(−)

Nearly all models included measures of vegetation configuration, hydrology, elevation, agriculture, and human influence. To create the binary habitat maps, we used cutoff values of 0.530 and 0.349, which corresponded to the averaged maximum sensitivity plus specificity thresholds for the state- and local-scale Maxent models, respectively. Using these thresholds, we identified 45,703 km^2^ of habitat at the state-scale and 23,798 km^2^ of local habitat, and, of these areas, 17,160.94 km^2^ were identified as habitat by both models (Figs. 2 and 3a, Table 3).

**Table 3:** Amount (km^2^), and percent of black bear habitat in each bear management unit as identified by local- and state-scale consensus habitat suitability consensus models, with a threshold of the maximum sensitivity plus specificity values as identified by Maxent (0.349 and 0.530, respectively).

	Big Bend BMU	Central BMU	Eastern Panhandle BMU	North BMU	South BMU	South Central BMU	Western Panhandle BMU	Total
Local-scale model	483.68 (2%)	10,253.16 (43%)	4415.34 (19%)	870.15 (4%)	3419.3 (14%)	2603.18 (11%)	1704.82 (7%)	23,749.63
State-scale model	5126.14 (11%)	11,010.42 (24%)	10,307.06 (23%)	3903.69 (9%)	5003.73 (11%)	7985.75 (17%)	2358.03 (5%)	45,694.82

The largest habitat patches identified by both scales were located around the Apalachicola subpopulation in the Eastern Panhandle BMU, the Ocala subpopulation in the Central BMU, and the South Florida subpopulation in the South BMU. Within the local-scale model, the largest patches were 1512.4 km^2^, 1960.0 km^2^, 3011.7 km^2^, respectively. The largest patches identified in the state-scale model measured 4130.1 km^2^, 2497.60 km^2^ and 1571.50 km^2^, respectively (Figs. 1 and 3a).

### Threats and protection

Under a worst-case 305 cm sea level rise scenario, our models identified a combined total of 5428.80 km^2^ of state and local black bear habitat area which may be lost due to inundation. Of this total area, 43.29% was habitat identified by both state and local models. Of all local habitat, 13.88% may be lost, and 9.8% of state habitat may be inundated (Table 4, Fig. 3b). However, the best-case, 30 cm scenario, predicted ~ 1% of both state and local habitat could be affected. With a business-as-usual development scenario, 15.57% of all state habitat, and 6.81% of local habitat may be lost to development. Of the 8733.25 km^2^ habitat identified as possibly under development threat by state and local models combined, 32.05% was identified as habitat under development pressure by both models (Fig. 3c). When overlaid with county, state, and federally protected lands, we found 53.64% of state habitat, 14.55% of local habitat were unprotected, and 25.69% of all unprotected habitat combined was identified by both state and local models as unprotected. Of the area of unprotected habitat, 27.63% of state habitat and 43.29% local habitat overlaps with projected development. Under the worst-case sea level rise scenario, 6.54% and 1.20% of unprotected state and local habitats could be inundated, respectively (Fig. 3d).

**Table 4:** Amount (km^2^) and percent of total respective areas of Florida black bear habitat identified local- and state-scale habitat models, that may overlap two sea level rise scenarios, projected development, and area unprotected.

Threat	Local model	State model
Habitat (km^2^) flooded at 30 cm sea level rise	226.67 (0.95%)	3298.44 (13.89%)
Habitat flooded with 305 cm sea level rise	19.70 (1.14%)	4480.27 (9.8%)
Habitat overlapping projected development	1616.86 (6.81%)	7116.39 (15.57%)
Unprotected habitat	3456.46 (14.55%)	24,511.99 (53.64%)

In total, local-scale models identified 23,749.63 km^2^ and state-scale models identified 45,694.82 km^2^ of black bear habitat throughout Florida.

## Discussion

Our models provide the first comprehensive, statewide habitat distribution model for black bears in Florida. We created models using some of the best practices for SDMs, taking into account scale and spatial biases. Our results provide robust insights for statewide and local conservation efforts, with high accuracy. The local-scale models had higher accuracies, with a tradeoff of identifying less habitat than the state-scale models (Table 3). In general, we found high habitat suitability along the Gulf Coast of Florida, along the eastern edge of Florida, and throughout south Florida, with differences in environmental predictors at the state- and local-scales.

The State of Florida has been focused on female black bear conservation and their role in population expansion, due to their philopatric nature and low reproductive rates^106^. Most of the GPS collar data collected and used here was from female bears, and we therefore had more female than male locations in our models (about a 5:1 ratio), likely giving rise to a female bias in the habitat suitability we identified. While it is important to identify potential habitat for females, we expand the knowledge of Florida black bear preferences by including males, and we recommend identifying movement habitat for dispersing male bears in any future connectivity work. In addition, species distribution models assume independent samples, and though we took steps to reduce autocorrelation and bias within our data, we recognize these models may still be biased towards specific individuals or subpopulations.

### Local-scale models

The variables in the top subpopulation models varied (Table 2), reflecting the general habitat requirements of black bears, their behavioral plasticity, and the different environments across Florida in which bears are found. Our results indicated that suitable bear habitat varied by location and included both natural areas and areas of high human influence. In some areas suitability likely does not represent preference; bear subpopulations existed while the human population and development expanded, with bears adapting behaviorally to their modified environment. In some subpopulations, Maxent and Mahalanobis models had opposite results, depending on relationships with individual variables. We interpret this as moderate suitability for that area, and support for our use of a consensus model. For example, north of the Tampa Bay area, in the Big Bend BMU, suitable bear habitat included areas farther from agriculture and low primary roads density, but also areas closer to tertiary roads, cities, and higher population densities (Fig. 3a). These seemingly conflicting suitabilities likely reflected bears’ relatively restricted range. Bears are limited to the locally protected areas contiguous with the Chassahowitzka Wildlife Management Area near the coast in this BMU and would need to cross a major state highway eastward to access agricultural areas (Fig. 3a).

Differences can be seen across subpopulation habitats when comparing central Florida subpopulations with those in the northern and southern parts of the state. Suitable habitat for bears in central Florida included areas of high agricultural density and areas close to cities. These bears were located in a series of natural areas with abundant waterways, agriculture, and primary roads. Central Florida has a high human population density, and over half of occurrences of human-bear conflicts are reported from this area^13^. Bears may be attracted to neighborhoods with abundant food sources in this region, and our models indicated that bear habitat here was relatively abundant but appears fragmented, with larger protected lands bisected by highways. However, in the low human density areas of northern and southern Florida, natural areas were key components of bear habitat at the local-scale models (Fig. 3a). Here, habitat included areas farther from human development and high in natural vegetation contiguity. Both of these subpopulations occupied part of the large protected areas (Fig. 3a) and habitat suitability reflected the low-elevation natural areas that are common in these areas.

### State-scale model

As expected, the state-scale models were more general and identified more habitat, more evenly dispersed across Florida (Table 3, Figs. 2 and 3a). Distance to natural vegetation had the highest impact on habitat suitability. Across the state, areas of higher habitat suitability were located closer to natural vegetation, farther from roads, in areas with higher agriculture density and moderate wetland density, and farther from rivers. Statewide, only five variables contributed to the Maxent model, while the Mahalanobis model identified three additional variables (Table 2). Accuracies were slightly lower than the local-scale model, which may in part be due to differences in habitat among subpopulations. If several subpopulations have different habitat associations, as in Eglin and Osceola, for example^40^, fewer variables may have similar values amongst all subpopulations and thus fewer variables may be able to explain habitat suitability. As seen by the TSS, sensitivity, and specificity measures, the state-scale models were better at identifying habitat than discerning non-habitat. This is likely related to the more general nature of these models, and the fact that we were unable to obtain true absence data for accuracy assessment. However, because this is a growing and expanding black bear population, identifying habitat that is currently unoccupied but suitable is important for management and outreach considerations. In these areas, the state-scale model could be used as a general guide, and conservation of specific areas could then be informed by local-scale models. This underscores the need for a multiscale modeling effort, which can identify habitat unique to particular subpopulations.

### Effects of different scales and extents

The use of models at different scales allowed us to identify factors contributing to habitat distribution at both statewide and subpopulation scale which otherwise would have been missed. While we prepared environmental variables at different scales, we did not restrict the types of variables or alter the resolution at either scale in either model. In preliminary model testing, we restricted models to variables describing local and state conditions, but model accuracy improved when we allowed inclusion of any variable. Our model accuracies show that our variable selection process was thorough enough to describe environmental associations across scales.

We did not account for spatial bias in the local-scale models because environmental conditions within BMUs were more similar than across the state-scale, and we wanted to capture potential bear habitat across BMUs that could be important for future conservation. However, although slight, the higher accuracies in the local-scale models may in part be due to this difference in spatial sampling and extent^107^.

### Effects of consensus modeling

However robust, these results, like all presence-only SDM results, should be interpreted with caution. Results represent potential habitat suitability, not occupancy or habitat selection^30^. All habitat suitability models have pros and cons^80^, and averaging multiple models can reduce uncertainty^31^. We believe this to be the case in this study, with our individual model results providing different habitat distributions. Maxent and Mahalanobis calculate suitability differently and thus identify different areas as suitable, often either over- or under-predicting habitat^68,83,87^. There are other methods in combining multiple SDMs to improve model outcome, but our models have consistently high accuracy and we recommend considering model averaging in future modeling efforts, especially when dealing with wide-ranging carnivores.

### Effects of threshold selection

There are many ways in which thresholds are selected to display a continuous SDM output as a binary habitat/non-habitat result^108^. In choosing a threshold selection method in this study, we aimed to maximize the probability of true positives (sensitivity) and negatives (specificity) and to ensure that future conservation efforts included all areas where bears may be located, while reducing conservation costs by discriminating low-likelihood areas of suitability. The maximum sensitivity plus specificity threshold has been shown to successively discriminate between true presence and random locations, is independent of species prevalence^102^, and we suggest its use in future efforts when a binary threshold is desired. We recognize that black bears use a wide range of habitats, and we recommend using these thresholds only as guides.

### Threats and protection

Globally, sea levels are likely to rise 0.3–1.2 m by 2100^109^. Given the relatively low elevation of Florida and the concentration of bears near coastal areas (Fig. 3b), bear habitat could be further restricted by sea level rise in the near future. We identified 13.88% of local-scale habitat and 9.80% of state-scale habitat in areas of inundation under a worst-case ~ 3 m sea level rise scenario (Table 4). While this may be a liberal projection, we did not account for storm surges, which may have an even more severe impact on habitat quality and distribution and should be taken into account during planning efforts in this system^110^.

Not only will bears be more restricted geographically by sea level rise, but 15.57% of state and 6.81% of local bear habitat overlaps with projected development (Fig. 3c). There are county-wide development plans in place, with attempts to curtail further sprawl, but effectiveness of these plans is unclear^44^. While the bear population in Florida is currently expanding^23^, their available habitat is decreasing, which could lead to a future where human-bear conflicts increase, support for bear conservation decreases, and a bear population in parts of Florida that stabilizes or even decreases.

Despite these threats, a large amount of bear habitat is protected (Fig. 3d). The lowest amount of protection we found was among the habitat identified only by the state-scale model. State-scale habitat could be critical in future dispersal and immigration/emigration among subpopulations, as the population in general continues to rebound. There appears to be a lack of protection for this type of habitat in the area between Tallahassee and Gainesville (Fig. 3d) and this area could provide important movement corridors to bears in the Apalachicola and Osceola/Ocala subpopulations. Considering the swift development rate in Florida, natural lands that are not protected are likely vulnerable to development.

Unfortunately, the lack of protection and potential black bear habitat losses are not unique in the Anthropocene, and are often more severe for carnivore habitat globally^111^. For example, Bengal tiger (*Panthera tigris bengalensis*) habitat in the Sundarbans is likely to be lost to sea level rise by 2070^112^, central Sumatran tigers (*Panthera tigris sumatrae*) may lose > 50% of their habitat by 2050^113^, summer polar bear (*Ursus arctos*) habitat may decline by 68% by 2090–2099^114^, and in southern California development will reduce puma (*Puma concolor*) natal den sites by 20% by 2065^115^. Despite declining global populations and habitats, carnivores can be resilient and adapt to modified environments. Recent efforts to model future habitat and responses to development can provide managers with the tools to mitigate habitat loss or degradation and promote coexistence^116–119^. Conservation of carnivore populations globally will require clear and effective communication by scientists and the support of communities and governments. This may be more realistic in some areas than others. In much of the US, communities are beginning to recognize the value of intact ecosystems, including sustainable carnivore populations. Fortunately, the Florida black bear has been prioritized for conservation by the State of Florida, and populations have already increased since their historic low in the 1970s^19^. With continued support, mitigation of projected habitat loss, identification of landscape corridors using the best available science from various initiatives, and effective communication with communities and urban planners, black bear populations can continue to grow and expand.

### Conclusions and management recommendations

Continued development could have significantly negative consequences on wildlife globally^111^ and identifying habitat of species vulnerable to anthropogenic impacts should be one of the first steps in conservation and landscape planning. Subsequent connectivity planning across landscapes can increase persistence probabilities for fragmented populations or subpopulations^8^. While sprawl in the U.S. seems to be beginning to decrease^120^, that is not the case in Florida, where the human population is growing and wildlife habitat is increasingly fragmented due to anthropogenic impacts and/or climate change.

We provide these results to guide landscape conservation for Florida black bears, and this research underscores the point that while species may recover in population size and distribution, conservation efforts should not wane in the face of projected human population growth and development. In Florida, we suggest that managers focus generally on wetland areas at higher elevation, particularly in unprotected areas in the South Central and Central BMUs. Areas that are isolated based on functional connectivity and bear dispersal abilities should be identified and conserved to maintain and/or create corridors. It is imperative that the remaining subpopulations are connected to allow sustainable bear population growth and improved genetic exchange as outlined in the 2019 Florida Black Bear Management Plan^19^. Without continuing statewide habitat conservation based on these results, the population increases and range expansion of the Florida black bear may stall before subpopulations are fully reconnected or exceed the social carrying capacity of the area. Carnivores are at risk, and our results serve as a reminder that even species that are considered recovered may face future threats to conservation without adequate habitat conservation.

## Supplementary information

Supplementary Information.
