# Supplementary material for: Multiscale consensus habitat modeling for landscape level conservation prioritization
Source: Sci Rep. 2020 Oct 20;10:17783. doi: 10.1038/s41598-020-74716-3 (PMC7576151; doi:10.1038/s41598-020-74716-3)
Supplement: Supplementary file 1 — Supplementary Information. [file 41598_2020_74716_MOESM1_ESM.docx]

**Multiscale consensus habitat modeling for landscape level conservation prioritization**

Erin E. Poor, Brian K. Scheick, and Jennifer M. Mullinax

**Supplementary Material**

**Table 1**

Environmental variables that were tested for each maximum entropy Florida black bear habitat suitability model

| **Variable** | **Source** | **Methods** |
| --- | --- | --- |
| Natural veg.^1^ distance | Florida Cooperative Land Cover v. 3.3; 2018 | Euclidean distance |
| Natural veg. contiguity | Florida Cooperative Land Cover v. 3.3; 2018 | McGarigal et al. 2012 |
| Natural veg. shape area index | Florida Cooperative Land Cover v. 3.3; 2018 | McGarigal et al. 2012 |
| Density of creeks, rivers, canals | NHD (1:12,000); 1988-2012 | 0.5 km radius circular moving window |
| River density | NHD (1:12,000); 1988-2012 | 0.5 km radius circular moving window |
| Population density | Gridded Population of the World; 2015 | NA |
| Dist. to cities | National Atlas of the United States; 2004 | Euclidean distance |
| Density of primary and secondary roads | TIGER/Line; 2016 | 0.5 km radius circular moving window |
| Density of tertiary roads | TIGER/Line; 2016 | 0.5 km radius circular moving window |
| Elevation | National Elevation Dataset; 2016 |  |
| TRI | National Elevation Dataset; 2016 | Riley et al. 1999 |
| Density of (freshwater forest/shrub) wetland | National Wetlands Inventory v. 2; 1977-2018 | 0.5 km radius circular moving window |
| Dist. to agriculture | USDA National Agriculture Statistics Service; 2016 | Euclidean distance |
| Agriculture density | USDA National Agriculture Statistics Service; 2016 | 0.5 km radius circular moving window |
| Dist. to food/denning habitat^2^ | Florida Cooperative Land Cover v. 3.3; 2018 | Euclidean distance |
| Local density of food/denning habitat | Florida Cooperative Land Cover v. 3.3; 2018 | 0.5 km radius circular moving window |
| Neighborhood density of food/denning habitat | Florida Cooperative Land Cover v. 3.3; 2018 | 1.5 km radius moving window; Clark et al. 2015 |

^1^Natural vegetation land cover categories from Florida Cooperative Landcover (FNAI 2018): Bare soil/clear cut, basin swamp, baygall, coastal scrub, coastal strand, clearcut wetlands, cypress, cypress/tupelo, dome swamp, dry flatwoods, dry prairie, floodplain marsh, floodplain swamp, freshwater forested wetlands, freshwater non-forested wetlands, high pine and scrub, hydric hammock, isolated freshwater marsh isolated freshwater swamp, mangrove swamp, maritime hammock, marshes, mesic flatwoods, mesic hammock, mixed hardwood-coniferous, natural rivers and streams, other coniferous wetlands, other hardwood wetlands, palmetto prairie, pine flatwoods and dry prairie, pine rockland, prairies and bogs, riverine, rockland hammock, sand pine scrub, sandhill, scrub, scurb mangrove, scrubby flatwoods, shrub and brushland, slope forest, strand swamp, tree plantations, upland glade, upland hardwood forest, upland pine, wet coniferous plantations, wet flatwoods, xeric hammock (italicized categories are from the FNAI Coop site land cover)

^2^Forage vegetation land cover categories from Florida Cooperative Landcover (FNAI 2018): Alluvial forest, basin swamp, baygall, bottomland forest, cabbage palm, cabbage palm flatwoods, cabbage palm hammock, coastal hydric hammock, coastal scrub, cutthroat grass flatwoods, cypress, cypress/tupelo, dome swamp, dry flatwoods, dry prairie, dry upland hardwood forest, gum pond, hydric hammock, hydric pine flatwoods, hydric pine savanna, isolated freshwater swamp, live oak, mesic flatwoods, mesic hammock, mixed hardwoods, oak scrub, palmetto prairie, pine-mesic oak, pine flatwoods and dry prairie, pond pine, prairie hydric hammock, prairie mesic hammock, rosemary scrub, sand pine scrub, scrubby flatwoods, shrub bog, south Florida bayhead, strand swamp, successional hardwood forest, titi swamp, tupelo, upland hardwood forest, upland mixed woodland, wet flatwoods, xeric hammock

**Table 2.** Accuracy assessment measures, area under the curve (AUC), the Boyce Index, true skill statistics (TSS), sensitivity, and specificity for 10 cross-validation runs of Maxent and Mahalanobis distance Florida black bear (*Ursus americanus floridanus*) habitat suitability models throughout the Big Bend Bear Management Unit (BMU).

|  |  | Big Bend BMU | | | | | | |  |  |
| --- | --- | --- | --- | --- | --- | --- | --- | --- | --- | --- |
| Model Number | Maxent | | | | | Mahalanobis | | | | |
|  | AUC | Boyce | TSS | Sensitivity | Specificity | AUC | Boyce | TSS | Sensitivity | Specificity |
| 1 | 0.92 | 0.95 | 0.86 | 0.94 | 0.92 | 0.93 | 0.94 | 0.77 | 0.84 | 0.93 |
| 2 | 0.92 | 0.99 | 0.86 | 0.93 | 0.93 | 0.96 | 0.95 | 0.76 | 0.82 | 0.94 |
| 3 | 0.93 | 0.99 | 0.86 | 0.94 | 0.92 | 0.96 | 0.95 | 0.77 | 0.84 | 0.93 |
| 4 | 0.91 | 0.99 | 0.86 | 0.92 | 0.95 | 0.96 | 0.94 | 0.76 | 0.80 | 0.95 |
| 5 | 0.90 | 0.99 | 0.86 | 0.94 | 0.92 | 0.94 | 0.94 | 0.77 | 0.83 | 0.94 |
| 6 | 0.92 | 0.99 | 0.86 | 0.93 | 0.93 | 0.96 | 0.97 | 0.76 | 0.83 | 0.93 |
| 7 | 0.93 | 0.94 | 0.85 | 0.95 | 0.90 | 0.95 | 0.95 | 0.78 | 0.85 | 0.92 |
| 8 | 0.91 | 0.92 | 0.86 | 0.93 | 0.94 | 0.94 | 0.95 | 0.76 | 0.81 | 0.94 |
| 9 | 0.92 | 0.98 | 0.86 | 0.94 | 0.92 | 0.95 | 0.96 | 0.77 | 0.84 | 0.93 |
| 10 | 0.91 | 0.99 | 0.85 | 0.90 | 0.95 | 0.93 | 0.97 | 0.75 | 0.79 | 0.96 |
| AVE | 0.92 | 0.97 | 0.86 | 0.93 | 0.93 | 0.95 | 0.95 | 0.76 | 0.83 | 0.94 |
|  |  |  |  |  |  |  |  |  |  |  |

**Table 3.** Accuracy assessment measures, area under the curve (AUC), the Boyce Index, true skill statistics (TSS), sensitivity, and specificity for 10 cross-validation runs of Maxent and Mahalanobis distance Florida black bear (*Ursus americanus floridanus*) habitat suitability models throughout the Central Bear Management Unit (BMU).

|  |  | Central BMU | | | | | | |  |  |
| --- | --- | --- | --- | --- | --- | --- | --- | --- | --- | --- |
| Model Number | Maxent | | | | | Mahalanobis | | | | |
|  | AUC | Boyce | TSS | Sensitivity | Specificity | AUC | Boyce | TSS | Sensitivity | Specificity |
| 1 | 0.79 | 0.98 | 0.46 | 0.83 | 0.63 | 0.83 | 0.98 | 0.47 | 0.69 | 0.78 |
| 2 | 0.81 | 0.99 | 0.45 | 0.80 | 0.66 | 0.82 | 1.00 | 0.46 | 0.65 | 0.81 |
| 3 | 0.80 | 0.98 | 0.45 | 0.77 | 0.68 | 0.82 | 0.98 | 0.45 | 0.63 | 0.82 |
| 4 | 0.80 | 0.99 | 0.45 | 0.79 | 0.67 | 0.82 | 0.99 | 0.46 | 0.65 | 0.81 |
| 5 | 0.79 | 0.99 | 0.45 | 0.79 | 0.67 | 0.83 | 0.96 | 0.46 | 0.65 | 0.81 |
| 6 | 0.81 | 1.00 | 0.45 | 0.79 | 0.67 | 0.83 | 0.99 | 0.46 | 0.65 | 0.81 |
| 7 | 0.79 | 1.00 | 0.46 | 0.83 | 0.63 | 0.82 | 0.99 | 0.47 | 0.69 | 0.78 |
| 8 | 0.80 | 0.99 | 0.46 | 0.83 | 0.63 | 0.82 | 0.98 | 0.47 | 0.68 | 0.79 |
| 9 | 0.80 | 0.99 | 0.45 | 0.78 | 0.67 | 0.82 | 0.98 | 0.46 | 0.64 | 0.82 |
| 10 | 0.80 | 1.00 | 0.46 | 0.83 | 0.64 | 0.82 | 0.98 | 0.47 | 0.68 | 0.78 |
| AVE | 0.80 | 0.99 | 0.46 | 0.80 | 0.65 | 0.82 | 0.98 | 0.46 | 0.66 | 0.80 |
|  |  |  |  |  |  |  |  |  |  |  |

**Table 4.** Accuracy assessment measures, area under the curve (AUC), the Boyce Index, true skill statistics (TSS), sensitivity, and specificity for 10 cross-validation runs of Maxent and Mahalanobis distance Florida black bear (*Ursus americanus floridanus*) habitat suitability models throughout the Eastern Panhandle Bear Management Unit (BMU).

|  |  | Eastern Panhandle BMU | | | | | | |  |  |
| --- | --- | --- | --- | --- | --- | --- | --- | --- | --- | --- |
| Model Number | Maxent | | | | | Mahalanobis | | | | |
|  | AUC | Boyce | TSS | Sensitivity | Specificity | AUC | Boyce | TSS | Sensitivity | Specificity |
| 1 | 0.83 | 0.98 | 0.58 | 0.85 | 0.73 | 0.92 | 1.00 | 0.69 | 0.79 | 0.90 |
| 2 | 0.83 | 0.99 | 0.55 | 0.79 | 0.77 | 0.93 | 1.00 | 0.67 | 0.75 | 0.91 |
| 3 | 0.85 | 0.99 | 0.54 | 0.75 | 0.79 | 0.93 | 0.99 | 0.65 | 0.73 | 0.92 |
| 4 | 0.84 | 0.96 | 0.58 | 0.85 | 0.73 | 0.93 | 0.99 | 0.69 | 0.79 | 0.90 |
| 5 | 0.84 | 0.96 | 0.58 | 0.84 | 0.74 | 0.93 | 1.00 | 0.68 | 0.78 | 0.90 |
| 6 | 0.83 | 0.97 | 0.57 | 0.81 | 0.76 | 0.93 | 0.99 | 0.67 | 0.75 | 0.91 |
| 7 | 0.84 | 0.98 | 0.58 | 0.83 | 0.75 | 0.93 | 1.00 | 0.68 | 0.77 | 0.91 |
| 8 | 0.84 | 0.99 | 0.54 | 0.76 | 0.78 | 0.93 | 1.00 | 0.66 | 0.74 | 0.92 |
| 9 | 0.82 | 0.96 | 0.57 | 0.82 | 0.75 | 0.93 | 1.00 | 0.67 | 0.77 | 0.91 |
| 10 | 0.84 | 0.99 | 0.55 | 0.78 | 0.77 | 0.93 | 0.99 | 0.66 | 0.75 | 0.91 |
| AVE | 0.84 | 0.98 | 0.56 | 0.81 | 0.76 | 0.93 | 0.99 | 0.67 | 0.76 | 0.91 |

**Table 5.** Accuracy assessment measures, area under the curve (AUC), the Boyce Index, true skill statistics (TSS), sensitivity, and specificity for 10 cross-validation runs of Maxent and Mahalanobis distance Florida black bear (*Ursus americanus floridanus*) habitat suitability models throughout the North Panhandle Bear Management Unit (BMU).

|  |  | North BMU | | | | | | |  |  |
| --- | --- | --- | --- | --- | --- | --- | --- | --- | --- | --- |
| Model Number | Maxent | | | | | Mahalanobis | | | | |
|  | AUC | Boyce | TSS | Sensitivity | Specificity | AUC | Boyce | TSS | Sensitivity | Specificity |
| 1 | 0.83 | 0.98 | 0.83 | 0.97 | 0.86 | 0.92 | 1.00 | 0.79 | 0.84 | 0.95 |
| 2 | 0.83 | 0.99 | 0.84 | 0.97 | 0.87 | 0.93 | 1.00 | 0.79 | 0.83 | 0.95 |
| 3 | 0.85 | 0.99 | 0.84 | 0.97 | 0.87 | 0.93 | 0.99 | 0.79 | 0.84 | 0.95 |
| 4 | 0.84 | 0.96 | 0.83 | 0.94 | 0.89 | 0.93 | 0.99 | 0.77 | 0.81 | 0.96 |
| 5 | 0.84 | 0.96 | 0.83 | 0.96 | 0.87 | 0.93 | 1.00 | 0.78 | 0.83 | 0.95 |
| 6 | 0.83 | 0.97 | 0.83 | 0.98 | 0.85 | 0.93 | 0.99 | 0.80 | 0.85 | 0.95 |
| 7 | 0.84 | 0.98 | 0.83 | 0.95 | 0.88 | 0.93 | 1.00 | 0.78 | 0.82 | 0.96 |
| 8 | 0.84 | 0.99 | 0.83 | 0.95 | 0.88 | 0.93 | 1.00 | 0.78 | 0.82 | 0.95 |
| 9 | 0.82 | 0.96 | 0.83 | 0.97 | 0.86 | 0.93 | 1.00 | 0.79 | 0.84 | 0.95 |
| 10 | 0.84 | 0.99 | 0.83 | 0.95 | 0.88 | 0.93 | 0.99 | 0.77 | 0.82 | 0.96 |
| AVE | 0.84 | 0.98 | 0.83 | 0.96 | 0.87 | 0.93 | 0.99 | 0.78 | 0.83 | 0.95 |

**Table 6**. Accuracy assessment measures, area under the curve (AUC), the Boyce Index, true skill statistics (TSS), sensitivity, and specificity for 10 cross-validation runs of Maxent and Mahalanobis distance Florida black bear (*Ursus americanus floridanus*) habitat suitability models throughout the South Central Panhandle Bear Management Unit (BMU).

|  |  | South Central BMU | | | | | | |  |  |
| --- | --- | --- | --- | --- | --- | --- | --- | --- | --- | --- |
| Model Number | Maxent | | | | | Mahalanobis | | | | |
|  | AUC | Boyce | TSS | Sensitivity | Specificity | AUC | Boyce | TSS | Sensitivity | Specificity |
| 1 | 0.88 | 1.00 | 0.83 | 0.96 | 0.87 | 0.93 | 0.98 | 0.71 | 0.81 | 0.90 |
| 2 | 0.88 | 0.98 | 0.81 | 0.98 | 0.83 | 0.93 | 0.99 | 0.71 | 0.84 | 0.88 |
| 3 | 0.87 | 0.97 | 0.84 | 0.93 | 0.91 | 0.93 | 1.00 | 0.69 | 0.76 | 0.94 |
| 4 | 0.87 | 0.98 | 0.82 | 0.96 | 0.86 | 0.93 | 0.99 | 0.71 | 0.81 | 0.90 |
| 5 | 0.87 | 0.98 | 0.84 | 0.94 | 0.90 | 0.91 | 0.99 | 0.70 | 0.77 | 0.93 |
| 6 | 0.88 | 0.99 | 0.83 | 0.95 | 0.88 | 0.92 | 1.00 | 0.71 | 0.79 | 0.92 |
| 7 | 0.87 | 0.97 | 0.84 | 0.91 | 0.92 | 0.93 | 1.00 | 0.67 | 0.73 | 0.95 |
| 8 | 0.88 | 0.99 | 0.84 | 0.94 | 0.89 | 0.91 | 0.99 | 0.70 | 0.77 | 0.92 |
| 9 | 0.88 | 0.98 | 0.83 | 0.96 | 0.87 | 0.93 | 0.99 | 0.71 | 0.81 | 0.91 |
| 10 | 0.87 | 0.99 | 0.83 | 0.96 | 0.87 | 0.94 | 0.99 | 0.71 | 0.80 | 0.91 |
| AVE | 0.87 | 0.98 | 0.83 | 0.95 | 0.88 | 0.93 | 0.99 | 0.70 | 0.79 | 0.91 |

**Table 7.** Accuracy assessment measures, area under the curve (AUC), the Boyce Index, true skill statistics (TSS), sensitivity, and specificity for 10 cross-validation runs of Maxent and Mahalanobis distance Florida black bear (*Ursus americanus floridanus*) habitat suitability models throughout the South Panhandle Bear Management Unit (BMU).

|  |  | South BMU | | | | | | |  |  |
| --- | --- | --- | --- | --- | --- | --- | --- | --- | --- | --- |
| Model Number | Maxent | | | | | Mahalanobis | | | | |
|  | AUC | Boyce | TSS | Sensitivity | Specificity | AUC | Boyce | TSS | Sensitivity | Specificity |
| 1 | 0.82 | 1.00 | 0.78 | 0.92 | 0.86 | 0.90 | 0.98 | 0.62 | 0.74 | 0.88 |
| 2 | 0.82 | 0.98 | 0.78 | 0.92 | 0.86 | 0.89 | 0.98 | 0.62 | 0.74 | 0.88 |
| 3 | 0.83 | 1.00 | 0.78 | 0.92 | 0.85 | 0.90 | 0.99 | 0.64 | 0.76 | 0.87 |
| 4 | 0.82 | 1.00 | 0.78 | 0.91 | 0.87 | 0.89 | 0.95 | 0.61 | 0.72 | 0.89 |
| 5 | 0.83 | 1.00 | 0.77 | 0.94 | 0.83 | 0.89 | 0.98 | 0.66 | 0.80 | 0.86 |
| 6 | 0.82 | 0.99 | 0.77 | 0.89 | 0.89 | 0.91 | 0.98 | 0.57 | 0.67 | 0.90 |
| 7 | 0.83 | 0.99 | 0.78 | 0.91 | 0.87 | 0.89 | 0.99 | 0.61 | 0.72 | 0.89 |
| 8 | 0.82 | 0.99 | 0.78 | 0.89 | 0.88 | 0.90 | 0.98 | 0.59 | 0.70 | 0.90 |
| 9 | 0.83 | 0.99 | 0.78 | 0.90 | 0.88 | 0.90 | 0.99 | 0.60 | 0.71 | 0.89 |
| 10 | 0.82 | 0.99 | 0.78 | 0.93 | 0.84 | 0.91 | 0.98 | 0.65 | 0.79 | 0.87 |
| AVE | 0.82 | 0.99 | 0.78 | 0.91 | 0.86 | 0.90 | 0.98 | 0.62 | 0.74 | 0.88 |

**Table 8.** Accuracy assessment measures, area under the curve (AUC), the Boyce Index, true skill statistics (TSS), sensitivity, and specificity for 10 cross-validation runs of Maxent and Mahalanobis distance Florida black bear (*Ursus americanus floridanus*) habitat suitability models throughout the Western Panhandle Bear Management Unit (BMU).

|  |  | Western Panhandle BMU | | | | | | |  |  |
| --- | --- | --- | --- | --- | --- | --- | --- | --- | --- | --- |
| Model Number | Maxent | | | | | Mahalanobis | | | | |
|  | AUC | Boyce | TSS | Sensitivity | Specificity | AUC | Boyce | TSS | Sensitivity | Specificity |
| 1 | 0.91 | 0.87 | 0.77 | 0.96 | 0.81 | 0.85 | 0.82 | 0.57 | 0.87 | 0.70 |
| 2 | 0.91 | 0.91 | 0.76 | 0.97 | 0.79 | 0.86 | 0.92 | 0.55 | 0.89 | 0.66 |
| 3 | 0.89 | 0.95 | 0.73 | 0.98 | 0.75 | 0.86 | 0.95 | 0.55 | 0.92 | 0.63 |
| 4 | 0.92 | 0.97 | 0.76 | 0.87 | 0.88 | 0.86 | 0.91 | 0.55 | 0.72 | 0.83 |
| 5 | 0.90 | 0.98 | 0.77 | 0.95 | 0.82 | 0.85 | 0.86 | 0.58 | 0.85 | 0.73 |
| 6 | 0.91 | 0.93 | 0.77 | 0.90 | 0.86 | 0.87 | 0.89 | 0.57 | 0.76 | 0.80 |
| 7 | 0.92 | 0.95 | 0.77 | 0.94 | 0.83 | 0.88 | 0.96 | 0.59 | 0.83 | 0.76 |
| 8 | 0.92 | 0.97 | 0.77 | 0.89 | 0.87 | 0.87 | 0.87 | 0.56 | 0.74 | 0.82 |
| 9 | 0.91 | 0.95 | 0.78 | 0.91 | 0.87 | 0.85 | 0.91 | 0.58 | 0.77 | 0.81 |
| 10 | 0.92 | 0.97 | 0.78 | 0.92 | 0.85 | 0.86 | 0.89 | 0.58 | 0.80 | 0.79 |
| AVE | 0.91 | 0.95 | 0.76 | 0.93 | 0.83 | 0.86 | 0.90 | 0.57 | 0.82 | 0.75 |

**Table 9.** Accuracy assessment measures, area under the curve (AUC), the Boyce Index, true skill statistics (TSS), sensitivity, and specificity for 10 cross-validation runs of Maxent and Mahalanobis distance Florida black bear (*Ursus americanus floridanus*) habitat suitability models throughout the state of Florida.

|  |  | Statewide | | | | | | |  |  |
| --- | --- | --- | --- | --- | --- | --- | --- | --- | --- | --- |
| Model Number | Maxent | | | | | Mahalanobis | | | | |
|  | AUC | Boyce | TSS | Sensitivity | Specificity | AUC | Boyce | TSS | Sensitivity | Specificity |
| 1 | 0.76 | 1.00 | 0.36 | 0.81 | 0.55 | 0.71 | 0.99 | 0.31 | 0.77 | 0.54 |
| 2 | 0.76 | 1.00 | 0.36 | 0.81 | 0.55 | 0.71 | 0.99 | 0.31 | 0.77 | 0.53 |
| 3 | 0.76 | 1.00 | 0.35 | 0.77 | 0.59 | 0.72 | 0.98 | 0.31 | 0.75 | 0.56 |
| 4 | 0.77 | 1.00 | 0.36 | 0.79 | 0.57 | 0.71 | 0.99 | 0.31 | 0.76 | 0.55 |
| 5 | 0.76 | 1.00 | 0.36 | 0.79 | 0.57 | 0.71 | 0.97 | 0.31 | 0.76 | 0.55 |
| 6 | 0.76 | 1.00 | 0.36 | 0.81 | 0.55 | 0.71 | 0.99 | 0.31 | 0.77 | 0.53 |
| 7 | 0.76 | 1.00 | 0.36 | 0.79 | 0.57 | 0.71 | 0.98 | 0.31 | 0.76 | 0.55 |
| 8 | 0.76 | 1.00 | 0.36 | 0.81 | 0.55 | 0.71 | 0.98 | 0.31 | 0.77 | 0.53 |
| 9 | 0.76 | 1.00 | 0.35 | 0.77 | 0.59 | 0.71 | 0.99 | 0.31 | 0.75 | 0.56 |
| 10 | 0.77 | 1.00 | 0.36 | 0.79 | 0.57 | 0.71 | 0.99 | 0.31 | 0.76 | 0.55 |
| AVE | 0.76 | 1.00 | 0.36 | 0.79 | 0.57 | 0.71 | 0.99 | 0.31 | 0.76 | 0.55 |
